# Immunological Outcome in Haploidentical-HSC Transplanted Patients Treated with IL-10-Anergized Donor T Cells

**DOI:** 10.3389/fimmu.2014.00016

**Published:** 2014-01-31

**Authors:** Rosa Bacchetta, Barbarella Lucarelli, Claudia Sartirana, Silvia Gregori, Maria T. Lupo Stanghellini, Patrick Miqueu, Stefan Tomiuk, Maria Hernandez-Fuentes, Monica E. Gianolini, Raffaella Greco, Massimo Bernardi, Elisabetta Zappone, Silvano Rossini, Uwe Janssen, Alessandro Ambrosi, Monica Salomoni, Jacopo Peccatori, Fabio Ciceri, Maria-Grazia Roncarolo

**Affiliations:** ^1^Division of Regenerative Medicine, Stem Cells and Gene Therapy, San Raffaele Scientific Institute, San Raffaele Telethon Institute for Gene Therapy, Milan, Italy; ^2^Hematology and BMT Unit, San Raffaele Hospital, Milan, Italy; ^3^TcLand Expression S. A., Nantes, France; ^4^Miltenyi Biotec GmbH, Bergisch-Gladbach, Germany; ^5^MRC Center for Transplantation, King’s College London, London, UK; ^6^Unit of Immunohaematology and Transfusion Medicine Service, San Raffaele Hospital, Milan, Italy; ^7^Center for Statistics in Biomedical Sciences, San Raffaele Scientific Institute, Milan, Italy; ^8^MolMed SpA, Milan, Italy; ^9^Vita-Salute San Raffaele University, Milan, Italy

**Keywords:** hematopoietic stem cell transplantation, haploidentical, IL-10, T regulatory type 1 cells, cell therapy, tolerance

## Abstract

T-cell therapy after hematopoietic stem cell transplantation (HSCT) has been used alone or in combination with immunosuppression to cure hematologic malignancies and to prevent disease recurrence. Here, we describe the outcome of patients with high-risk/advanced stage hematologic malignancies, who received T-cell depleted (TCD) haploidentical-HSCT (haplo-HSCT) combined with donor T lymphocytes pretreated with IL-10 (ALT-TEN trial). IL-10-anergized donor T cells (IL-10-DLI) contained T regulatory type 1 (Tr1) cells specific for the host alloantigens, limiting donor-vs.-host-reactivity, and memory T cells able to respond to pathogens. IL-10-DLI were infused in 12 patients with the goal of improving immune reconstitution after haplo-HSCT without increasing the risk of graft-versus-host-disease (GvHD). IL-10-DLI led to fast immune reconstitution in five patients. In four out of the five patients, total T-cell counts, TCR-Vβ repertoire and T-cell functions progressively normalized after IL-10-DLI. These four patients are alive, in complete disease remission and immunosuppression-free at 7.2 years (median follow-up) after haplo-HSCT. Transient GvHD was observed in the immune reconstituted (IR) patients, despite persistent host-specific hypo-responsiveness of donor T cells *in vitro* and enrichment of cells with Tr1-specific biomarkers *in vivo*. Gene-expression profiles of IR patients showed a common signature of tolerance. This study provides the first indication of the feasibility of Tr1 cell-based therapy and paves way for the use of these Tr1 cells as adjuvant treatment for malignancies and immune-mediated disorders.

## Introduction

The long-term benefit and wide applicability of T-cell-depleted (TCD) haploidentical-HSC transplantation (haplo-HSCT), have been limited by slow immune reconstitution and high relapse rate (1–6). Infusion of unmanipulated donor lymphocyte (DLI) to improve immune reconstitution and to prevent relapse led to high incidence of severe graft-versus-host disease (GvHD) (3, 7).

During the last decade, considerable progress has been made on the use of *ex vivo* manipulated T cells as a new advanced therapeutics to promote graft-versus-leukemia (GvL) and immune reconstitution without GvHD after HSCT (8, 9). For example, chimeric antigen-receptor modified T cells to directly treat leukemia or suicide-gene expressing T cells have been successfully employed (9–12). In addition, freshly isolated or expanded CD4^+^CD25^+^ T regulatory (Treg) cells have been used as adjuvant therapy to suppress GvHD after allogeneic HSCT in patients with hematologic malignancies (13–15). These Treg cell therapies are safe, although tested in a small number of patients, but their efficacy is still controversial. In addition, many open questions remain on the best source of Treg cells to be administered, the optimal cell dose, the survival and behavior of these cells once they are in the host environment, and their mechanism of action.

Peripheral T regulatory type 1 (Tr1) cells, with alloantigen (Allo-Ag)-specific suppressor function, have been consistently associated to a state of tolerance in chimeric patients after allogeneic HSCT. In addition, several preclinical studies demonstrated that Tr1 cells play a major role in the induction and maintenance of transplantation tolerance (16–21). Tr1 cells are characterized by a unique cytokine production profile (IL-10^+^IL-4^−^IL-17^−^) (17, 21), and by the co-expression of CD49b and LAG-3 (22). Tr1 cells control immune responses by secreting high levels of IL-10, and by killing myeloid cells (17, 23). Allo-Ag-specific Tr1 cells can be induced *in vitro* in the presence of IL-10 (17, 24, 25). Activation of T cells by Allo-Ags in the presence of IL-10 induces Ag-specific hypo-responsiveness, identified as T-cell anergy (26, 27). These IL-10-anergized T cells contain Tr1 cells, as demonstrated at single-cell level (17), but also memory T cells able to proliferate in response to nominal Ags, such as tetanus toxoid and *Candida albicans*, and viral Ags, such as Epstein-Barr virus (25). In a murine model of allogeneic HSCT, transfer of IL-10-anergized T cells prevented GvHD (28). These findings provided the rationale for the clinical use of IL-10-anergized donor T cells (IL-10-DLI) to support immune reconstitution without severe GvHD after allo-HSCT.

We developed a GMP-validated protocol to obtain IL-10-DLI, from haploidentical hematopoietic stem cell donors (25, 29). In a small proof-of-concept clinical trial (ALT-TEN trial), 12 patients who received TCD haplo-HSCT were treated with IL-10-DLI. Here, we describe the positive outcome of TCD haplo-HSCT combined with IL-10-DLI cell therapy in four patients with high-risk/advanced stage hematologic malignancies. These patients achieved full donor chimerism, immune reconstitution, and long-term persistent disease remission. Cells from all four patients expressed biomarkers consistent with tolerance and Tr1 cell enrichment. This study indicates the feasibility of cell therapy with IL-10-DLI to promote fast immune reconstitution and tolerance after haplo-HSCT.

## Materials and Methods

### Patients and transplant procedures

**Figure 1:**
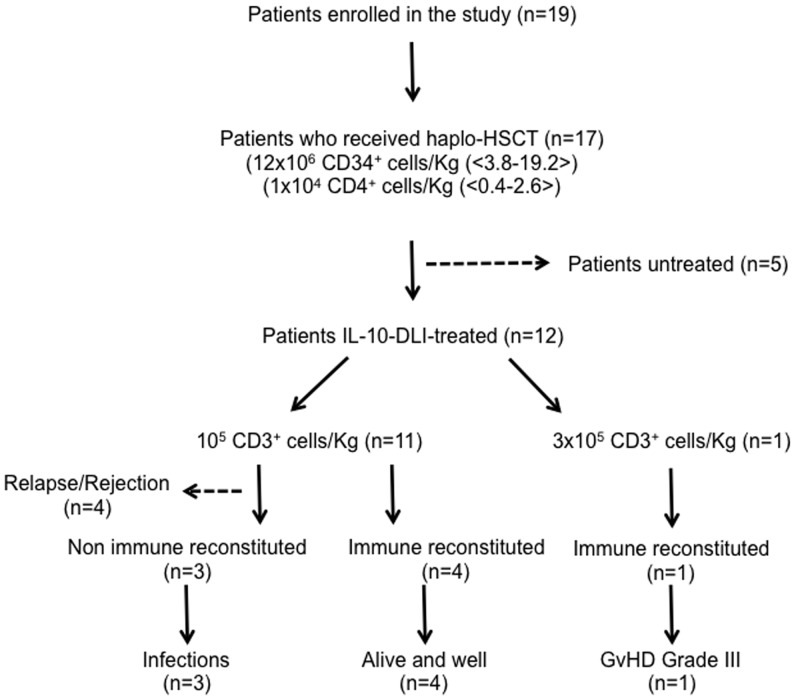
**Patients’ distribution and overall outcome of patients enrolled in ALT-TEN trial**. IL-10-DLI: IL-10-anergized donor T cells; immune reconstituted patients: CD3^+^ T cells >100/μl.

Nineteen patients with high-risk hematopoietic malignancies, median age 39 years (range: 20–64), were enrolled from August 2000 to October 2009 in a single center, non-randomized prospective phase I–II clinical trial (ALT-TEN protocol) approved by the San Raffaele Scientific Institute’s Ethics Committee and the Italian Ministry of Health. The clinical trial was registered in the public cell therapy national database of the Italian Istituto Superiore di Sanità (registration number IS/11/6172/8309/8391). All patients signed informed consent. Enrolled patients were candidates for haplo-HSCT from family donors, since they did not have a timely, suitable HLA-matched family or unrelated donor. Full haplotype mismatching was defined as ≥2 HLA-A, HLA-B, or DRB1 antigen disparity. At the time of trial design, information from the literature and our clinical experience clearly indicated that overall probability of survival after TCD haplo-HSCT was very poor (1). Therefore, the approval for the ALT-TEN trial, as a proof-of-concept study, was given for a small cohort of patients, and the San Raffaele Scientific Institute’s Ethics Committee agreed on a single-arm study design (see Table 1 for overall patients’ characteristics, treatment, and outcome). Patients received myeloablative conditioning and standard infection prophylaxis with voriconazole, levofloxacine, or acyclovir, followed by infusion of highly purified granulocyte-colony-stimulating (G-CSF)-mobilized CD34^+^ donor cells, selected with the CliniMACS one-step GMP-validated procedure (CliniMACS, Miltenyi Biotec, Bergisch-Gladbach, Germany), containing approximately 10^4^ CD3^+^/kg donor-derived T cells. Time to myeloid engraftment was defined as the first of three consecutive days of neutrophil counts ≥500/μl and platelet counts ≥25000/μl. Complete chimerism was defined as ≥95% of donor cells in the bone marrow. The distribution of patients enrolled in the trial is reported in Figure 1. Two (patients 8 and 17) of the 19 patients enrolled in the proof-of-concept ALT-TEN clinical trial were not transplanted because of relapse in the pre-conditioning phase and donor screening failure, respectively, thus they were excluded from the study because they did not undergo haplo-HSCT. Transplanted patients (*n* = 17) received a median of 12 × 10^6^ CD34^+^ cells/kg (range, 3.8–19.2), and 1 × 10^4^ CD3^+^ T cells/kg (range, 0.4–2.6). All but two transplanted patients (patients 12 and 19, in whom primary graft failed), achieved myeloid engraftment. Median time to an absolute neutrophil count of 500/μl and to a platelet count >25000/μl was 15 days (range, 10–23 and 10–26, respectively; patient 2 never reached a platelet count >25000/μl). IL-10-DLI were successfully good-manufacturing-practice (GMP)-produced in 14/17 patients (patients 10, 13, and 19: level of anergy <67%). Among these 14 patients, 2 could not be treated with IL-10-DLI because of graft failure (patient 12), or because they did not meet the eligibility criterion for early autologous reconstitution (patient 15 and patient 13, who also had no anergy). Therefore, five of the transplanted patients did not receive IL-10-DLI. Among them, two died because of infections (patients 10 and 12 at day 169 and 32 post-HSCT, respectively), two had disease recurrence (patients 13 and 15), and one (patient 19) received a second transplant from the same haploidentical donor because of graft failure.

**Table 1:** **Outcome of the IL-10-DLI treated patients**.

Pt No.	Sex/ age	Disease (status at haplo-HSCT)	Myeloid engraftment		IR	aGvHD (grade)	GvHD treatment duration (months)	Outcome (cause)	Overall survival
							
			PMN	PLTs					
								
			Days from haplo-HSCT		Days from IL-10-DLI				Days from haplo-HSCT
1	M/21	HD (Advanced)	18	18	no	no	–	Death (infection)	43
2	F/59	sAML (Advanced)	13	?	no	no	–	Death (relapse)	72
3	M/31	NHL (Advanced)	17	26	no	no	–	Death (infection)	97
4	M/39	ALL (Advanced)	10	10	no	no	–	Death (relapse)	35
5	F/36	AML (CR3)	11	11	18	24 (III)	11	Death (infection)	355
6	M/58	NHL (Advanced)	23	24	15	47(II/III)	39	Alive/IR	3017
7	M/64	AML (PIF)	13	15	28	34 (II)	4	Alive/IR	2653
9	M/20	HD (Advanced)	19	18	102	99 (II)	10	Alive/IR	2441
11	F/22	AML (CR2)	17	14	33	74 (II)	1.5	Alive/IR	2339
14	M/29	HD (Advanced)	12	20	no	no	–	Death (infection)	69
16	F/25	HD (Advanced)	22	22	no	no	–	Death (graft rejection)	44
18	M/39	AML (CR1)	17	17	no	no	–	Death (graft rejection)	43

*Pt No., patient number of enrollment. M, male; F, female diseases: HD, Hodgkin’s disease; sAML, secondary acute myeloid leukemia; NHL, non-Hodgkin lymphoma; ALL, acute lymphoblastic leukemia; AML, acute myeloid leukemia; MDS, myelodisplastic syndrome; Haplo-HSCT, hematopoietic stem cell transplantation from haploidentical donors. As “advanced status of diseases” are considered lymphoprolipherative disorders in partial remission or with progressive disease and acute leukemia not in remission. CR, complete remission (CR1 first, CR2 second, CR3 third); PIF, primary induction failure; PMN, polymorphonucleated cells; PLTs, platelets; IR, Immune reconstitution; CD3^+^ T cells >100/μl. IL-10-DLI, IL-10-anergized donor T cells. For patient 9, who received 2 IL-10-DLI, timing of immune reconstitution and acute GvHD onset is calculated from the first IL-10-DLI. aGVHD, acute graft-versus-host-disease*.

### IL-10-anergized T-cell production and protocol of infusion

PBMC were collected by leukapheresis from the donors before G-CSF mobilization, and from the patients before the conditioning regimen. Cell manipulation was performed in GMP-validated conditions and cell product manufacture was approved by the Italian Ministry of Health. The method of cell production and the characterization of the cell product have been described in detail (25, 29). The donor-derived cell product was obtained after a mixed lymphocyte reaction (MLR) toward host-derived cells, in the presence of IL-10 (clinical-grade provided by Schering-Plough), for 10 days (MLR/10) (25). Host-derived stimulator cells were CD3-depleted PBMC. The cell product acquired host-specific hypo-responsiveness, called “anergy” and calculated as follows: [MLR – MLR/10 : MLR] × 100. Data obtained in preclinical studies showed that IL-10-anergized cells do not change their phenotype as compared to the unmanipulated donor PBMC, do express very low activation markers such as CD25 and HLA-DR, are anergic toward the host alloAgs in proliferation and have a decreased frequency of (antigen specific) cytotoxic CD8^+^ T cell precursors.

Percentages of anergy (≥67%, calculated as above and based on mean values −2 SD of percentage of anergy obtained from more than 30 donor pairs during the process of development) and cell viability (≥70%) were chosen as release criteria because considered to be essential for safety and efficacy of the infused cells. Cell viability, phenotype, anergy, and proliferative capacity of each of the cell preparations infused in the patients showed no significant variability and are reported in the Table 2.

**Table 2:** **Immunophenotype and function of IL-10-DLI infused in patients of the ALT-TEN trial**.

Markers	Treated (*n* = 12)		IR (*n* = 5)		Non-IR (*n* = 7)	
	mean	SD	mean	SD	mean	SD
**Immunophenotype (% of positive cells in the lymphocyte gate)**						
CD2	78.6	12.4	79.4	4.0	77.9	17.1
CD3	74.0	13.2	76.8	7.3	71.7	17.0
CD4	55.8	14.7	56.3	11.0	55.4	18.4
CD8	32.6	13.0	33.0	9.4	32.2	16.3
CD14	17.4	12.0	23.3	14.6	12.5	7.4
CD56	26.0	13.8	32.7	17.0	20.5	8.5
CD19	18.0	10.8	19.0	11.2	17.2	11.5

**Stimulation**	**Treated** **(*n* = 12)**		**IR** **(*n* = 5)**		**Non-IR** **(*n* = 7)**	
	**mean**	**SD**	**mean**	**SD**	**mean**	**SD**

**Anergy (%)**						
Host-APC	76.8	7.7	80.4	9.3	74.1	5.6
**Proliferation (%)**						
PMA/iono	81.3	16.2	88.8	15.6	76.0	15.6

*Cell composition and host antigen-specific unresponsiveness in secondary MLR, named anergy, and polyclonal response (to PMA^+^ionomycin, PMA/iono) of IL-10-DLI prior to infusion. Percentages are calculated as percentage of proliferative responses of MLR/10 versus MLR cell cultures*.

*Mean values and standard deviations (SD) for the group of immune reconstituted (IR) and non-IR patients are reported. All cell products had vitality >80%*.

IL-10-DLI, either fresh or frozen, were administered after sustained neutrophil engraftment and not before 1 month post-HSCT in order to exclude confounding overlapping events, such as acute GvHD due to contaminating donor T cells comprised within the graft and lytic effect of ATG toward the IL-10-DLI. All infusions were performed in the absence of Immunosuppression (IS). Eligibility criteria for IL-10-DLI were lack of immune reconstitution, defined by circulating CD4^+^ T cells ≤50/μl, no signs of GvHD, and absence of relapse. The protocol was designed for administration of escalating cell doses every three patients (10^5^, 3 × 10^5^ and 10^6^, 3 × 10^6^ CD3^+^ T cells/kg), or of the dose not considered toxic in at least 10 patients.

The primary endpoints of the cell therapy trial were safety defined as no acute adverse reactions, no bone marrow aplasia and lack of GvHD > grade II unresponsive to treatment. Secondary endpoints were immune reconstitution, defined as peripheral CD3^+^ T cells >100/μl (value based on the time of effective immune reconstitution reported in previous trials of adoptive transfer performed at our Institute) (10), number of infectious episodes and restored *in vitro* immunological functions.

### Immunological studies

Routine tests, required during follow-up after haplo-HSCT, were performed at the HSR diagnostic laboratories (Laboraf), and included complete hematological evaluation, immunophenotyping (CD3, CD4, CD8, CD16, CD56, and CD19), monitoring of blood or tissue CMV and Epstein-Barr viral loads. Furthermore, immunophenotype analyses for surface detection of CD25 (2A3, BD Biosciences, San Diego, CA, USA), CD45RA (MI100, Biolegend, San Diego, CA, USA), CD62L (DREG 56, BD Biosciences, San Diego, CA, USA), CD24 (ML5, BD Biosciences, San Diego, CA, USA), CD38 (HIT2, BD Biosciences, San Diego, CA, USA), CD49b (AK-7, Biolegend, San Diego, CA, USA), LAG-3 (FAB2319P, R&D System) and intracytoplasmic detection of FOXP3 (259D, BioLegend, San Diego, CA, USA), GZ-B (GB11, Caltag MedSystems, Buckingham, UK), and phycoerythrin (PE)-labeled anti-IL-10 (JESS-19F1, BD Biosciences, San Diego, CA, USA) were performed on freshly or frozen isolated PBMC at HSR-TIGET. Human peripheral blood was obtained from healthy donors upon informed consent in accordance with local ethical committee approval (TIGET PERIBLOOD) and with the Helsinki Declaration. PBMC were isolated by centrifugation over Lymphoprep Ficoll gradients (Axis-Shield PoC AS, Oslo, Norway). For FOXP3 detection cells were fixed and permeabilized with FOXP3 Fix/Perm buffer set (Biolegend), stained following the manufacturer’s instructions and analyzed by FACS Calibur (BD). The staining for CD49b and LAG-3 was performed at 37°C for 15 min. Samples were acquired using a BD FACS Canto flow cytometer (BD Biosciences), and data were analyzed with FlowJo software. Quadrant markers were set accordingly to unstained controls.

For *in vitro* functional assays total PBMC were plated at 10^5^/well in 96-well plates in the presence of viral antigens: HSV-1 and -2 (2.5 μg/ml; Advanced Biotechnologies Inc., Columbia, USA) or CMV (lysate of infected human fibroblasts; diluted 1:30; kindly provided by Dr. Chiara Bonini, Laboratory of Experimental Hematology, HSR, Milan Italy). At day 4, cells were pulsed overnight with 1 μCi/well ^3^H-thymidine (Perkin Elmer, MA, USA). In parallel, cells were stimulated with coated anti-CD3 (10 μg/ml; OKT3; Ortoclone, Jansen-Cilag, Raritan, NJ, USA) and soluble anti-CD28 (1 μg/ml; BD Pharmingen, San Diego, CA, USA) mAbs, PHA (1 μg/ml; Roche Diagnostics GmbH, Mannheim, Germany), and IL-2 (50 UI/m; Chiron Italia, Milan, Italy) for 3 days. After stimulation, cells were pulsed for 16 h with 1 μCi/well ^3^H-thymidine. For IFN-γ ELISPOT analysis, multiScreen HTS 96-well plates (Millipore, Billerica, MA, USA) were coated with captured anti-human IFN-γ mAb (IFNγ ELIspot KIT, ENELHIFNG, Thermo Scientific, Rockford, IL, USA) (30, 31). After saturation, thawed PBMC from patients and healthy donors were added in triplicate wells (3 × 10^5^ cells/well) and were incubated for 18 h at 37°C in 5% CO_2_ in the presence of CMV infected cell lysate. Stimulation with PMA (10 ng/ml; Sigma, St Louis, MO, USA) plus Ionomycin (150 ng/ml; Sigma) was used as a positive control, and cells in medium alone were used as negative control. After washing, the second biotinylated anti-IFN-γ mAb (Thermo Scientific) was added, followed by Streptavidin–Alkaline Phosphatase conjugate (Roche) and then substrate (AEC Staining Kit, Sigma). After drying, spots were counted using an Elispot Reader (A. ELVIS GmbH, Hannover, Germany). The number of CMV-specific T cells, expressed as spot-forming cells per 1 × 10^6^ PBMC, was calculated after the subtraction of negative control values.

### T-cell repertoire analysis

TCR repertoire analysis (TcLandscape) was performed by TcLand Expression (Nantes, France), following the standardized and validated methods established in RISET [reprograming the immune system for the establishment of tolerance (RISET)^1^]. PBMC from patients’ blood, collected in EDTA tubes, were isolated by centrifugation over Lymphoprep Ficoll gradient (Axis-Shield) and frozen in EUROzol^®^ reagent (Euroclone Spa Life Sciences Division, Milan, Italy) for RNA extraction. Total RNA was reverse-transcribed using an Invitrogen-Life Technologies cDNA synthesis kit (Roche Diagnostics). Complementary DNA was amplified by PCR using couples of primers specific for each Vβ genes (32). The amplification products were then elongated and electrophorized on a capillary sequencer (Applied Biosystems – Life Technologies 3730) to separate them according to their length (33). The complementarity-determining-region-3 (CDR3) profiles or spectra types obtained were transformed into mathematical distributions. In parallel, the level of Vβ family transcripts was measured by real-time quantitative PCR and normalized by a housekeeping gene (HPRT). Each CDR3 length distribution was then multiplied by the corresponding normalized Vβ transcript amounts and combined within the TcLandscape^®^ representation (34). To explore the evolution of the TCR repertoire of a patient, we used the Principal Component Analysis (PCA) on the percentage of the Vβ family transcripts. This multivariant exploratory statistical technique is used to simplify complex data sets and to provide a “projection” of the data into a reduced, easily visualized space. In our context, PCA allowed us to project the different time-points of each patient in a factorial space defined by axes, named components (C), which are linear combination of optimally weighted observed variables. The PAST software (Palstat: Statistics for Paleontologists and Paleobiologists. JS Whalley, PD Ryan 1995) was used for PCA and data representation.

### Gene-expression analysis

Microarray analysis was carried out by Miltenyi Biotec GmbH (Bergisch-Gladbach, Germany), according to the validated RISET methods. The data discussed in this publication have been deposited in NCBI’s Gene-Expression Omnibus (35) (GSE21885^2^). Peripheral vein blood was drawn directly into PAXgene blood RNA tubes (QIAgen, Crawley UK) and extracted using the PAXgene Blood RNA Kit, which includes DNAse I treatment (QIAgen). The quality and integrity of RNA were determined using the Agilent RNA 6000 Nano Kit on the Agilent 2100 Bioanalyzer (Agilent Technologies). RNA was quantified by measuring A260 nm on an ND-1000 Spectrophotometer (NanoDrop Technologies). 0.5 μg total RNA was labeled using the Agilent Low RNA Input Linear Amp Kit (Agilent Technologies) as detailed in the “One-color microarray-based gene-expression analysis protocol” (version 5.5, part number G4140-90040). 0.6 μg Cy3-labeled fragmented cRNA was hybridized on the RISET 1.0 microarray platform according to the “One-color microarray-based gene-expression analysis protocol” (version 5.5, part number G4140-90040). The last washing step was performed with acetonitrile for 30 s. RISET 1.0 is a custom Agilent 8 K × 15 K 60mer oligonucleotide microarray comprising 4,610 probes represented in triplicates that is dedicated to transplantation research and was designed based on published and unpublished data provided by RISET consortium partners. Probe design was optimized for the detection of multiple transcript variants of a gene, on optimized hybridization properties of the probes, and avoiding cross-hybridization. Fluorescence signals of the hybridized Agilent Microarrays were detected using Agilent’s Microarray Scanner System (Agilent Technologies, Inc.). The Agilent Feature Extraction Software (FES v. 10.2.1.3/10.5.1.1) was used to read out and process the microarray image files. To determine differential gene-expression, FES derived data were further analyzed using the Rosetta Resolver gene-expression data analysis system (v. 7.2.2.0.0., Rosetta Inpharmatics LLC).

For time-course analysis, background-corrected signal intensity values were transformed to base two logarithms and quantile normalized. The open-source STEM (“Short Time-series Expression Miner v1.3.4”) software (36) was used with the default settings and the “No normalization/add 0” option. Expression profiles representing microarray probes with an increase or decrease expression level in the late-time samples (≥1 year after IL-10-DLI) were selected separately for each patient. An intersection of probes, identified for each patient, was applied for the generation of hierarchically clustered heat maps by using the MeV package (v. 4.5.1) (37). The lists of genes, with increased or decreased expression levels in the late-time samples of patients 7, 9, and 11, were analyzed for a significant enrichment of biological pathway annotation terms in comparison to the complete RISET 1.0 microarray configuration. Term-enrichment relative to the expected background distribution was scored using Fisher’s exact test with Benjamini-Hochberg correction. Annotations were derived from different sources, e.g., Gene Ontology (GO^3^), signaling pathway membership, sequence motifs, chromosomal proximity, literature keywords, and cell-specific marker genes. No relevant terms were returned for the genes downregulated in the late-time samples (not shown). Gene-expression pattern of 166 time-course sensitive microarray probes were used for the generation of median inter-experiment correlation coefficients between patient and healthy control-derived samples, analyzed according to the time-span after starting IS and after its discontinuation.

The microarray dataset including patients 7, 9, and 11 generated on the RISET 1.0 Agilent platform (GEO Series accession number GSE21885) was merged with the dataset obtained from the cohort of kidney transplant recipients studied by Indices of Tolerance (IOT) consortium (38) using the RISET 2.0 platform (GEO Series accession number GSE14655), considering only the 4610 reporters present on both platforms. Expression profiles of the 10 most discriminatory biomarkers of tolerance identified by the IOT study were extracted from the combined quantile normalized, log2-transformed dataset. From this data, pairwise Pearson correlation coefficients comparing the medians of each IOT patient (Tol-DF, tolerant drug-free; s-CNI stably immunosuppressed by calcineurin inhibitors (CNI); s-nCNI, stable under non-CNI; s-LP, stable under low dose prednisolone; HC, healthy controls) were calculated with each separate time-point from patients 7, 9, and 11.

## Results

### Long-term survival in transplanted patients treated with IL-10-DLI

In a pilot study, 12 patients affected by high-risk/advanced stage hematologic malignancies and previously treated with multiple lines of chemotherapy, were transplanted with haplo-HSCT and received IL-10-DLI after myeloid engraftment (ALT-TEN trial). IL-10-DLI were infused at a median of 35 days post-HSCT (range, 28–64), when no spontaneous immune reconstitution was detectable in the periphery. Cells were infused in the absence of pharmacological IS. IL-10-DLI cell dose was 10^5^ CD3^+^ T cells/kg in all but one patient (patient 5), who received 3 × 10^5^ CD3^+^ T cells/kg. Four patients (patients 6, 7, 9, and 11), who received IL-10-DLI at the dose of 10^5^ CD3^+^ T cells/kg at a median of 40 days post-HSCT (range, 31–64), survived beyond day 100 post-HSCT and immune reconstituted (IR) at a median of 28 days post-IL-10-DLI (range, 15–102 days, Table 1). At present, these four long-term IR patients are IS-free with an optimal performance status at a median follow-up of 7.2 years (range 6.4–8.3). All four patients have a stable full donor chimerism. No disease relapse occurred in these reconstituted patients, despite the fact that two patients presented an advanced status of the disease at the time of transplant. Moderate acute GvHD was observed (grade II/III in patient 6 and grade II in patients 7, 9, and 11) starting at a median of 60 days (range, 34–99) after IL-10-DLI. Acute GvHD completely resolved after conventional therapy administered for a relatively short period in patients 7, 9, and 11 (4, 10, and 1.5 months, respectively), whereas patient 6 required treatment for 39 months, and responded well to second-line therapy (Table 1).

Overall, IL-10-DLI was efficacious in 4 out of 12 patients, possibly because these four patients were free of events for a time period that was sufficient for immune reconstitution to occur. The remaining eight patients included three patients who died for infections early post-HSCT (day 43, 69, and 97 in patients 1, 14, and 3, respectively) and four patients who died because of relapse or lack of engraftment (patient 2, 4, 16, and 18) shortly after transplant. One patient (patient 5) received a higher dose of IL-10-DLI (3 × 10^5^ CD3^+^ T cells/kg), which led to rapid immune reconstitution, but the patient developed severe GvHD unresponsive to treatment and died 1 year post-HSCT because of recurrent infections related to IS therapy (Figure 1; Table 1).

With the exception of the five IR patients described above, the overall survival of the patients treated with TCD haplo-HSCT and IL-10-DLI was comparable to that of patients receiving TCD haplo-HSCT without DLI. This negative outcome is due to the lack of immune reconstitution (not-IR) and is in line with that reported in high-risk patients treated with TCD haplo-HSCT without a complementary T-cell adoptive immunotherapy (“naked”) (1–3). None of the patients who failed to achieve immune reconstitution developed signs of GvHD.

IL-10-DLI cellular products received by the IR or not-IR patients were comparable in terms of cell composition (proportion of T, B, and NK-cells). Although not statistically different, the percentages of CD14^+^ and CD56^+^ were higher in IR patients as compared to non-IR patients (23.3 ± 14.6 vs. 12.5 ± 7.4% of CD14^+^ cells and 32.7 ± 17 vs. 20.5 ± 8.5% of CD56^+^ cells in IR and non-IR patients, respectively). Overall vitality, degree of host-specific-unresponsiveness and ability to proliferate in response to polyclonal stimulation of IL-10-DLI cell products in both IR and non-IR patients were not statistically significant (Table 2).

### IL-10-DLI promoted fast immune reconstitution after TCD haplo-HSCT

**Figure 2:**
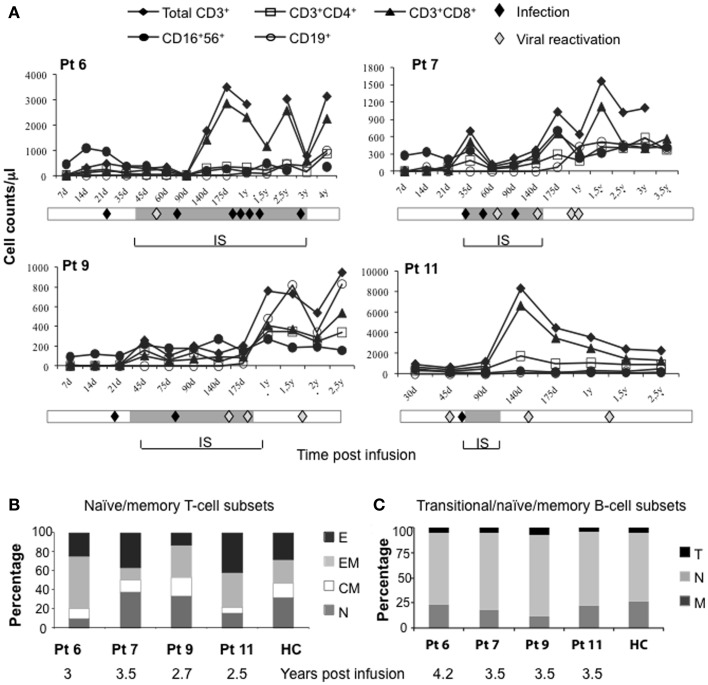
**Immune reconstitution in long-term-surviving IL-10-DLI patients**. (Pt 6, 7, 9, and 11). **(A)** Kinetics of recovery of peripheral lymphocyte subsets (absolute number), at different time-points (d, day; y, year) after infusion of IL-10-anergized donor T cells (IL-10-DLI). For patient 9, who received two subsequent IL-10-DLI, timing is calculated from the second IL-10-DLI. Reference values for healthy controls (HC) are: total lymphocytes 1000–4800/μl, CD3^+^ T cells 781–2664/μl, CD3^+^CD4^+^ T cells 397–1762/μl, CD3^+^CD8^+^ T cells 224–1762/μl, CD16^+^CD56^+^ NK-cells 56–690/μl, CD19^+^ B cells 57–710/μl. Gray bars indicate the time-frame of immunosuppression (IS). Infective episodes (febrile episodes of bacterial, fungal or unknown origin) and viral (cytomegalovirus, Varicella-Zoster virus, Herpes-Simplex virus, Hepatitis B virus) reactivations detected by molecular analysis prior to the appearance of clinical manifestations are indicated (filled and empty diamonds, respectively). **(B)** Relative distribution of naïve and memory in gated CD3^+^ T cells. E, effector T cells (CD62L^−^CD45RA^+^); EM, effector-memory T cells (CD62L^−^CD45RA^−^); CM, central memory T cells (CD62L^+^CD45RA^−^); N, naïve (CD62L^+^CD45RA^+^) T cells. HC, *n* = 20. **(C)** Relative distribution of B-cell subsets, according to the expression of CD24 and CD38 in gated CD19^+^ B-cells. T, transitional B cells (CD24brightCD38bright); N, naïve/mature B cells (CD24dullCD38^+^); M, memory B cells (CD24brightCD38^−^). HC, *n* = 3.

It is well established that after TCD haplo-HSCT complete functional restoration of T cells requires 6–9 months in pediatric recipients, and at least 1 year in adults (4, 5). In the four long-term surviving IL-10-DLI treated patients (patients 6, 7, 9, and 11), immune reconstitution occurred at a median of 30 days (range, 15–102) post-IL-10-DLI. At the time of immune reconstitution, the absolute number of CD3^+^ T cells was >250/μl (297, 394, 256, and 925 in patients 6, 7, 9, and 11, respectively), of CD3^+^CD4^+^ T cells was >150/μl (176, 163, 157, 294 in patients 6, 7, 9, and 11, respectively) and of CD3^+^CD8^+^ T cells was >100/μl (115, 231, 100, 631 in patients 6, 7, 9, and 11, respectively). Figure 2A shows the kinetics of natural killer (NK)-cell and T-cell recovery in peripheral blood. Peripheral NK cells repopulated shortly after IL-10-DLI (1–2 weeks). CD3^+^CD8^+^ T cells repopulated the peripheral blood before CD3^+^CD4^+^ T cells, leading to a persistent CD4/CD8 inverted ratio. The highest lymphocyte counts pre-GvHD were 1235, 1291, 500, and 1685 cells/μl, in patients 6, 7, 9, and 11, respectively. CD19^+^ B-cells were within normal limits (median, 98/μl; range, 72–251) at a median of 199 days (range, 150–276 days). These data show a faster peripheral lymphocytes repopulation in IL-10-DLI treated patients as compared to that reported in the literature for patients post-TCD haplo-HSCT (1). In these latter patients, NK cell recovery occurred in 2–4 weeks, whereas T-cell immune reconstitution was delayed, with CD4^+^ T cells <100 and <200/μl 10 and 16 months after transplantation, respectively, and B-cell reconstitution required up to 2 years (6). In IL-10-DLI treated IR patient, peripheral T lymphocytes had an effector-memory phenotype (CD45RA^−^CD62^−^), whereas naïve T cells (CD45RA^+^CD62L^+^) were detected 1 year post-IL-10-DLI (data not shown). At long-term follow-up, total lymphocyte counts were within normal range (2100–3200 cells/μl) (Figure 2A) with naïve/memory T cell subsets and transitional, naïve, and memory B-cell subsets all represented (Figures 2B,C).

### Normal TCR-Vbeta repertoire in IL-10-DLI treated patients

**Figure 3:**
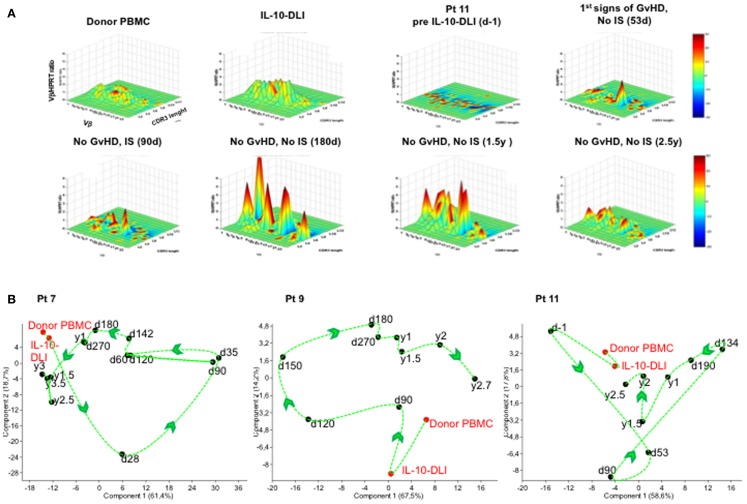
**TcLandscape^®^ prior and at different time-points after IL-10-DLI treatment in haplo-HSC transplanted patients**. T cell receptor (TCR) repertoires of the donor’s PBMC, IL-10-anergized donor T cells (IL-10-DLI) prior to infusion, and patient’s PBMC before and at different time-points post-IL-10-DLI, in the presence and absence of GvHD and immunosuppression (IS). **(A)** One representative patient (pt 11) out of four studied is shown. Four-dimensional graphs of the different Vβ families are shown. *X* axis, Vβ gene family; *y* axis, length of the complementarity- determining-region-3 (CDR3); *z* axis, amount of Vβ gene normalized by hypoxanthine-guanine phosphoribosyl transferase; color code, percentage of perturbation compared to normal Gaussian distribution; d, days; y, years. **(B)** TCR repertoire analysis. Evolution of Vβ gene-expression of each patient by Principal Component Analysis (PCA). PCA allows projection of the different time-points in a factorial space defined by axes, named “components,” which are linear combinations of optimally weighted observed variables. The distance between two time-points is related to their TcLandscape similarity. For each patient, the percentages of each Vβ family for all recipient time-points, were independently analyzed by PCA. The “itinerary” taken by the TCR repertoire of the patient during T-cell reconstitution is shown by a dashed curve and arrows. Pt: patient; IL-10-DLI: IL-10-anergized donor T cells infused into the patients; d: days; y: years.

The evolution of the TcLandscape demonstrated that in long-term follow-up IR patients, the TCR repertoire progressively became polyclonal and comparable to that of HC. The expression of the TCR-Vβ repertoires at different time-points during the follow-up is represented in Figure 3. Results in one representative patient (Figure 3A) show that the TCR repertoire in the IL-10-DLI cell product preinfusion was similar to that of the unmanipulated donor PBMC. We observed a flat landscape early post-HSCT, which indicates the absence of T cells. T-cell reconstitution started with olygoclonal T-cell expansion (red peaks in the landscape), corresponding to clinical signs of GvHD or infections (Figure 3A). Later on the TcLandscape gradually became polyclonal (green distributions) like the donor TcLandscape. The evolution of the TCR repertoire in the long-term follow-up patients (patients 7, 9, and 11) was also analyzed by PCA on each time-series of TcLandscapes performed (Figure 3B). PCA produces a map that summarizes the similarity of TCR-Vβ chain gene-expression considering recipient cells analyzed at all time-points, donor PBMC and IL-10-DLI TcLandscapes. It is noteworthy that donor PBMC and IL-10-DLI are close on the map indicating a similar TCR repertoire. The itinerary illustrates that the divergence between the donor T-cell repertoire and the repertoire of donor T cells post-haplo-HSCT decreases with time. The TCR of the recipient progressively resembles that of the donor, starting from a depleted T-cell landscape to a fully polyclonal T-cell repertoire.

### Immune functions are restored in patients with long-term survival

**Figure 4:**
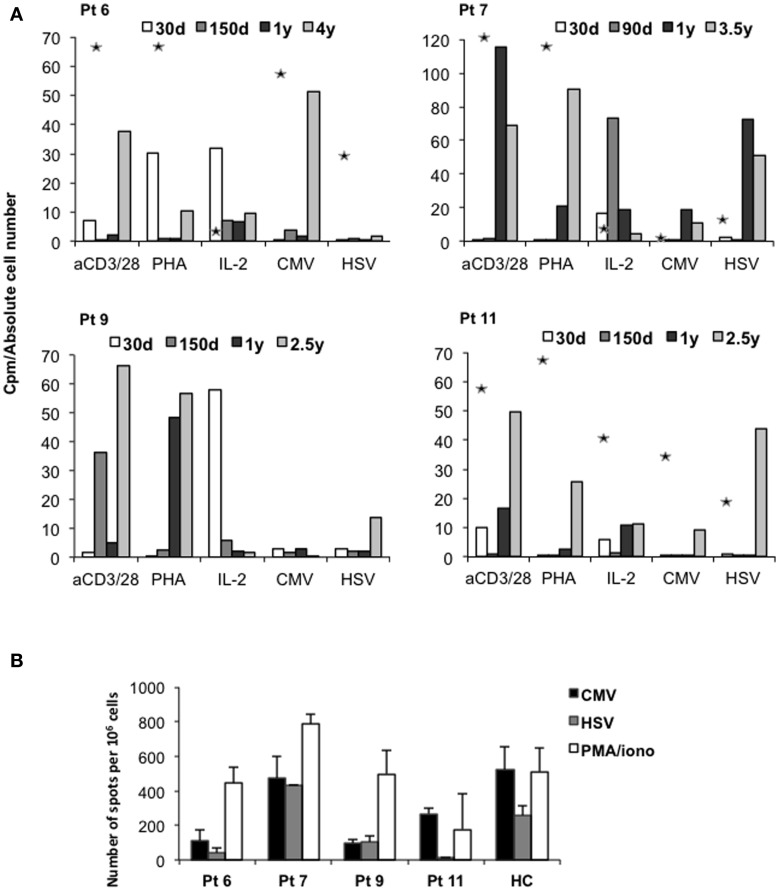
**Immune functions in long-term surviving IL-10-DLI treated patients**. **(A)** *In vitro* proliferative responses of PBMC to mitogens and specific antigens. Data are expressed as counts per minute (cpm) and are normalized for absolute number of lymphocytes: cpm obtained in response to addition of exogenous IL-2 are normalized to total lymphocyte counts. Cpm obtained in response to anti-CD3/CD28 (aCD3/CD28) monoclonal antibodies and other stimuli, phytohemagglutinin (PHA), and to the viral antigens Herpes-Simplex virus (HSV) and cytomegalovirus (CMV), are normalized to the number of CD3^+^ T cells. Asterisks indicate proliferative responses of haploidentical donor PBMC before transplant. Pt: patient; d: days; y: years. **(B)** Frequency of CMV- and HSV-specific T cells producing interferon (IFN)-γ in patients’ PBMC. Bars represent the number of IFN-γ producing T cells specific for CMV, HSV and PMA^+^ ionomycin (PMA/iono) in peripheral blood of patients (patients 6, 7, 9, and 11) tested 4, 3.5, 2.7, and 2.5 years after IL-10-DLI, respectively. Healthy controls (HC) were tested in parallel. Pt: patient.

*In vitro* proliferative responses to IL-2 were detectable early post-IL-10-DLI, consistent with the early reconstitution of NK cells. Proliferation in response to polyclonal stimuli [anti-CD3/CD28 monoclonal antibodies (mAb) or phytohemagglutinin (PHA)] and to viral antigens [CMV and Herpes-Simplex virus (HSV)] was detected between 6 and 12 months post-infusion, after immunosuppressive therapy withdrawal (Figure 4A). Interferon (IFN)-γ-producing CMV- and HSV-specific T cells (Figure 4B) were also detected by ELISPOT analysis, confirming the presence of viral reactive T cells in the peripheral blood of long-term IR patients. It should be noted that in historical controls *in vitro* T-cell function was still profoundly impaired 10 months after transplantation (1–3). Overall, even if in a limited number of patients, immune reconstitution in IL-10-DLI treated patients was consistently faster and more efficient in comparison to historical controls, with polyclonal and Ag-specific functional responses normalized within the first year.

Consistently with what observed *in vitro*, in long-term IR patients (patients 6, 7, 9, and 11) bacterial and fungal infections and viral reactivations mainly occurred during pharmacological IS (Figure 2A). Overall, in the first 100 days post-HSCT, the estimated median of infectious episodes was 2.75 episodes/patient in the IL-10-DLI treated long-term IR group of patients, whereas in the groups of the untreated or IL-10-DLI treated but non-IR patients, the estimated median of infectious episodes was approximately 4 episodes/patient. Importantly, no mortality related to infections was observed in the four IL-10-DLI treated IR patients, whereas the IL-10-DLI patients who did not achieve immune reconstitution died for infections and relapse at a median of 72 days from haplo-HSCT.

### IL-10-DLI immune reconstituted patients maintain host-Ag-specific unresponsiveness and display markers of IL-10-mediated tolerance

**Figure 5:**
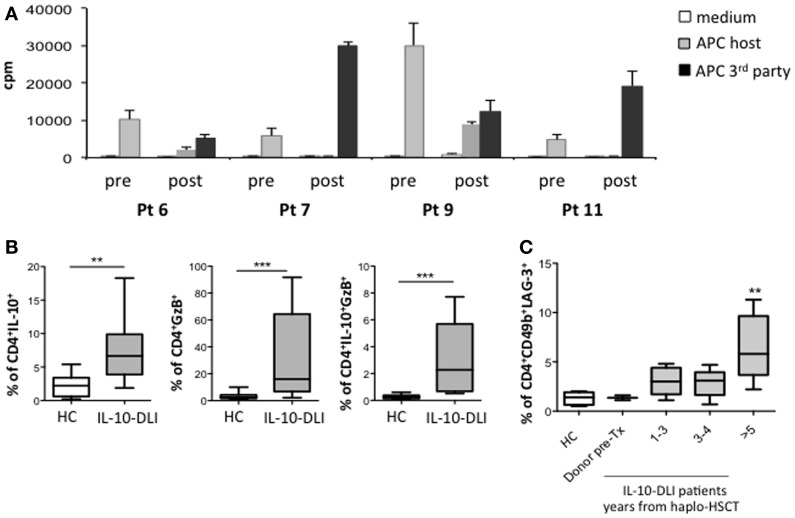
**Response to host allo-Ags and biomarkers of tolerance**. **(A)** Proliferative responses of donor-derived PBMC, toward host-monocytes before transplantation (pre). Proliferative responses of patient-derived PBMC (4, 3.5, 2.5, and 2.5 years, post-IL-10-DLI for pt 6, 7, 9, and 11, respectively) toward host-derived or third-party mature dendritic cells (post). **(B)** Intracytoplasmic granzyme-B (GZ-B) and IL-10 on gated CD4^+^ T cells from total PBMC of patients 6, 7, 9, and 11, tested 175 days, 2.8, 3, and 5 years; 180 days, 1.5, 2.5, 4, 5, and 7 years; 180 days, 1.5, 2, 2.7, 4.2, 5, and 6 years; 190 days, 1 year, 3.5, 4, 4.2, and 6 years after IL-10-DLI, respectively, and of healthy controls (HC) (HC: *n* = 10). Mean ± SD of the percentage of IL-10^+^ T cells of GzB^+^ T cells and of IL-10^+^GzB^+^ T cells in all time-points analyzed are shown, Differences were evaluated by means of the non-parametric Mann-Whitney *U* test. *P*-values < 0.05 were considered significant. **(C)** Surface CD49b and LAG-3 on gated CD4^+^ T cells from total PBMC of patients 6, 7, 9, and 11, tested 175 days, 2.8, 3, and 5 years; 180 days, 1.5, 2.5, 4, 5, and 7 years; 180 days, 1.5, 2, 2.7, 4.2, 5, and 6 years; 190 days, 1 year, 3.5, 4, 4.2, and 6 years after IL-10-DLI, respectively, and of HC (HC: n = 5), and of donor PBMC of pt 7 and 9 (Donor pre-Tx). Mean ± SD of the percentages of CD49b^+^LAG-3^+^ T cells at indicated time-points are shown. Differences were evaluated by means of the non-parametric Mann-Whitney *U* test. *P*-values < 0.05 were considered significant. cpm: counts per minutes of Tritiated Thymidine incorporation.

Donor-derived PBMC isolated from IR patients post-IL-10-DLI did not proliferate in response to host-antigen-presenting cells (APCs), in contrast to PBMC collected pre-transplant. However, these cells displayed normal responses to third party allo-APCs *in vitro* (Figure 5A). These results demonstrate the host-specific unresponsiveness of donor T cells after IL-10-DLI and immune reconstitution.

It is possible that host-specific unresponsiveness is maintained by the Tr1 cells infused with the IL-10-DLI cell product or by Treg cells *de novo* induced during IR. To test this latter hypothesis, we investigated the presence of CD4^+^CD25^+^FOXP3^+^ natural T regulatory cells (nTregs) and Tr1 cells in IR patients. Percentages of nTregs were comparable in patients and in HC (average 5.2 and 4.4% in patients and HC; respectively, not shown), whereas a low proportion of cells with the Treg-specific DNA demethylated region (TSDR) of forkhead-box-protein-3 (FOXP3) was detected in patients’ whole blood as compared to HC [average 0.49% in patients (range: 0.30–0.80%) and 1.4% in HC (range: 0.4–2.9%), in line with what previously described (39).

A significantly higher percentage of granzyme-B (GZ-B)^+^IL-10^+^ T cells (*P* = 0.0006) and of CD49b^+^ Lymphocyte Activation Gene 3 (LAG-3)^+^ T cells (*P* = 0.004) was detected in the PBMC of IL-10-DLI treated patients compared to HC (Figures 5B,C). Importantly, cells expressing these biomarkers progressively increased during follow-up. This observation is consistent with the high mRNA expression of GZ-B, and LAG-3, between 6 months and 3 years post-TCD haplo-HSCT in IL-10-DLI treated patients (not shown).

**Figure 6:**
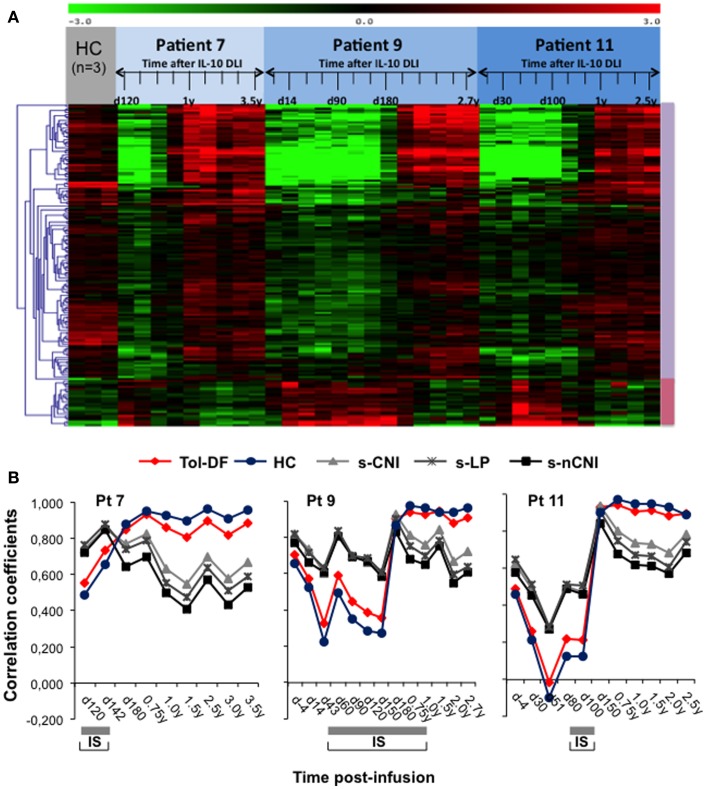
**Gene-expression profile analysis of three long-term follow-up patients**. **(A)** Matrix view of gene-expression data (heat map) showing genes with significantly increased (violet vertical bar) and decreased (pink vertical bar) expression levels in samples evaluated at different time-points post-IL-10-DLI (as indicated in days or years) of patient 7, patient 9, and patient 11 compared to three samples from healthy controls (HC). Data represent quantiles normalized and median-centered signal intensity data in log 2 space. Red and green indicate upregulation and downregulation, respectively, relative to the reporter-wise median. **(B)** Gene-expression analysis of the ten most discriminatory biomarkers of tolerance identified within the Indices of Tolerance (IOT) study in patients 7, 9, and 11. Pairwise Pearson correlation coefficients comparing the medians of expression in each IOT patient group (Tol-DF: tolerant drug-free; s-CNI: stably immunosuppressed by calcineurin inhibitors (CNI); s-nCNI: stable under non-CNI; s-LP: stable under low dose prednisolone; HC: healthy controls) with the expression in each separate time-point sample from patients 7, 9, and 11 are shown. The expression of the most discriminatory IOT biomarkers in IL-10-DLI treated patients progressively became super-imposable to that in Tol-DF patients and HC. The gray bars below each graph indicate immunosuppressive treatment.

In each patient, we compared the gene-expression profile at immune reconstitution, during GvHD and after immune-suppressive therapy discontinuation to that of three HC recruited in the “IOT” RISET-EU project (IOT^4^). Consistently with the immune-reconstitution, the overall patients’ gene-expression profiles gradually approximated those of the HC, as visualized in a hierarchically clustered matrix view of gene-expression (Figure 6A) and calculated by the inter-experiment correlation coefficients (>0.9). Of note, in the IL-10-DLI treated patients the gene-expression profile of the top 10 ranked genes recently identified as biomarkers of tolerance (38, 40) progressively became super-imposable to that of kidney transplanted recipients who did not reject the organ after IS withdrawal. Conversely, the gene-expression profile of the IL-10-DLI tolerant patients was different from that of transplanted patients under IS and from that of HC (Figure 6B). Notably, unlike in HC, in tolerant IL-10-DLI treated patients and tolerant kidney transplanted recipients, B-cell-related genes were highly expressed [Figure 6B and (38, 40)].

## Discussion

Patients with high-risk/advanced stage hematologic malignancies who received TCD haplo-HSCT, not followed by any kind of adoptive immunotherapy (“naked” haplo-HSCT) or followed by unmanipulated donor T-cell infusions, showed a very high rate of mortality because of infection and relapse or because of GvHD (3, 7). In the present study, 12 patients with high-risk/advance stage hematologic malignancies treated with TCD haplo-HSCT were infused with donor T cells anergized toward the host allo-Ags by IL-10. These IL-10-DLI contained both host-specific Tr1 cells and fully competent polyclonal T cells, which could down modulate host-reactivity and mediate effector responses, respectively (25). Of these 12, 4 patients had a positive outcome after TCD haplo-HSCT combined with IL-10-DLI, showing fast immune reconstitution with restoration of normal T naïve and memory cell subsets, progressive increase in cells positive for the Tr1 biomarkers and a tolerance signature. These IR tolerant patients are long survivors, in good performance status and remain completely disease-free at 7.2 years follow-up (median).

Patients treated with IL-10-DLI who did not immune reconstitute displayed post-transplant courses that resemble that of “naked” transplants. It remains unclear why in these patients IL-10-DLI infusions were not sufficient to sustain immune reconstitution and to prevent infections and relapse. Pre-existing poor clinical conditions and advanced disease severity at the time of transplant could account for the lack of positive response to IL-10-DLI treatment (3, 41).

In the 4 long-term surviving patients, the infusion of IL-10-anergized T cells shortly after engraftment of the myeloid compartment, correlated with an accelerated immune reconstitution with no relapse, only moderate and transient acute GvHD and no chronic GvHD. These data strongly support the notion that IL-10-DLI contributed to the overall success of the treatment. In line with this conclusion is the observation that once infused, the IL-10-DLI expanded, as demonstrated by the increase in NK-cells, CD8^+^, and CD4^+^ T cells, and by the progressive restoration of a polyclonal TCR-Vβ repertoire. However, the infused donor-derived T cells specific for the host Ags remained unresponsive *in vitro*. It has to be defined whether the control of host-reactivity is mediated by Tr1 cells present in the IL-10-DLI infusion, which survived long-term *in vivo*, or is due to *de novo* generated Tr1 cells. Using a combination of novel Tr1 biomarkers, we showed a progressive expansion of GZ-B (42, 43)/IL-10 and CD49b/LAG-3 (22) expressing CD4^+^ T cells in the peripheral blood. Importantly, the percentages of these cells were significantly higher in the IL-10-DLI long-term surviving patients [(4, 5, 6) and 6 years after TCD haplo-HSCT for patient 6, 7, 9, and 11, respectively] compared to healthy subjects. CD49b and LAG-3 are bonafide biomarkers of Tr1 cells, which are consistently detected in tolerant patients with stable mixed chimerism after allogeneic HSCT (22). These data provide evidence of the long-term presence of Tr1 cells in fully immune competent tolerant patients 5 years after haplo-HSCT and IL-10-DLI treatment. Expansion of CD4^+^CD25^+^FOXP3^+^ T cells was not observed at any time in long-term IR IL-10-DLI treated patients suggesting that nTregs did not have a major role in the immune reconstitution and *in vivo* immune modulation (44, 45).

Importantly, after HSCT, the gene-expression profile of IL-10-DLI treated patients was super-imposable to the tolerance signature of organ-transplanted patients with tolerant outcomes after IS withdrawal. Specifically, B-cell-related genes, associated with operational tolerance in stable kidney transplanted patients IS-free (38, 40) and in renal transplant recipient treated with regulatory macrophages (46) were observed in IL-10-DLI treated patients. This finding supports the hypothesis that IL-10-DLI treated patients have operational tolerance and provides evidence that the tolerance signature, once established, can be consistent across different clinical settings.

Results obtained in 4 haplo-HSCT transplanted patients who survived long-term after IL-10-DLI are promising especially considering that 2 of these patients were transplanted during disease relapse. However, the overall course of the trial highlights a number of limitations. In the majority of the IL-10-DLI treated patients relapse or infections occurred prior to or in time proximity with the infusion, when immunodeficiency was still severe. Therefore, the selection of patients may not have been optimal to reveal immune modulating effects of the Tr1 cells comprised in the IL-10-DLI cell product. In addition, it is possible that IL-10-DLI should have been administered earlier after transplantation, to allow parallel myeloid engraftment and immune reconstitution with better prevention of infections and relapse. Furthermore, the Tr1 cell frequency in the cell product was rather low as demonstrated *in vitro* by IL-10 ELIspot analysis (25). At the lowest effective dose, IL-10-DLI did not cause adverse effects and only moderate and transient GvHD. However, a threefold increase in cell dose provoked GvHD grade III-IV in one patient, suggesting that the ratio between effector cells and Tr1 cells was too high.

The variability in patient’s outcome was not due to differences in the anergic state of the infused IL-10-DLI since this parameter was very consistent among all treated patients. However, it cannot be excluded that certain immune genetic characteristics of some patient/donor pairs, such as the combination of HLA-Ags mismatches, rendered them additionally susceptible to post-HSCT complications. In addition, the phenotype of infused IL-10-DLI cells was similar in IR patients vs. those who did not, although the former received an IL-10-DLI infusion that contained a higher, although not significant, proportion of CD56^+^ and CD14^+^ cells. The higher proportion of CD56^+^ cells in the IL-10-DLI may have led to earlier and better protection against infections, and possibly prevent disease relapse, after infusion, allowing T-cell expansion. Similarly, the higher proportion of CD14^+^ cells may reflect a higher number of APC with tolerogenic properties that could have favor, once *in vivo*, Tr1 cell differentiation and tolerance. Therefore, a possible contribution of NK cells and CD14^+^ cells in favoring faster immune reconstitution cannot be ruled out.

Early immune reconstitution post-HSCT has also been achieved with DLI genetically engineered to express the thymidine kinase suicide-gene (TK-DLI) (10), CD8-depleted DLI (47), CD25-depleted DLI (48), and CTLA4-Ig-treated BM T cells (49, 50). The incidence of severe GvHD remained high in all but the TK-DLI clinical trial, in which GvHD was controlled by acyclovir treatment, targeting the suicide-gene. Moreover, in these trials relapse occurred in a relevant proportion of patients (10). Freshly isolated or *in vitro* expanded Treg cells (13–15) have been infused in allo-HSCT or haplo-HSCT recipients to prevent GvHD. In these studies a reduction, but not abrogation, of GvHD was observed compared to historical controls. In addition, it should be noted that Treg cells were detected in the peripheral blood of treated patients only shortly after infusion (14). A major difference between the Treg cell trials and the trial with IL-10-DLI is that, in the former, a pool of polyclonal non-Ag-specific cells was administered, whereas we used a cell product containing adaptive *in vitro* primed Ag-specific Tr1 cells. A different approach to administer Ag-specific Tr1 cells has been reported in a cell therapy trial in patients affected with Chron’s disease. In this trial OVA-specific IL-10 producing T cell clones were infused and subsequently re-challenged *in vivo* by feeding patients, with an egg-derived peptide. In this study safety and indirect evidences of clinical benefit were reported but no *in vivo* tolerance signature was observed (51).

Overall, the small proof-of-concept clinical trial described here demonstrates the feasibility of cell therapy with host-specific Tr1 cells and shows promising, although preliminary and uncontrolled, efficacy in 4 patients. Future phase I/II trials with larger cohorts of transplanted patients, homogeneous in terms of disease and treatment, are required to further prove the efficacy of this treatment. The present study opens the possibility of using Tr1 cells as adjuvant treatment also in organ transplantation, as planned for renal transplant recipient (The ONE study, European Union Funded Multinational Clinical Trial), and in other immune-mediated disorders, including inflammatory bowel diseases and type 1 diabetes.

## Author Contributions

Rosa Bacchetta and Maria-Grazia Roncarolo designed and coordinated the study, analysed the data and wrote the paper; Fabio Ciceri supervised the clinical study; Barbarella Lucarelli collected and analyzed data and wrote the paper; Claudia Sartirana performed immunomonitoring; Silvia Gregori contributed to cell production, Tr1 studies and to manuscript editing; Maria T. Lupo Stanghellini, Raffaella Greco, Massimo Bernardi, Jacopo Peccatori contributed to clinical monitoring; Patrick Miqueu, did TCR studies; Stefan Tomiuk and Uwe Janssen did microarray analysis; Maria Hernandez-Fuentes contributed to gene-expression analysis; Elisabetta Zappone contributed to study design and initial clinical monitoring; Alessandro Ambrosi did statistical analysis; Monica Salomoni supervised cell production.

## Conflict of Interest Statement

The authors declare that the research was conducted in the absence of any commercial or financial relationships that could be construed as a potential conflict of interest.

## Footnotes

^1^www.risetfp6.org

^2^http://www.ncbi.nlm.nih.gov/geo/query/acc.cgi?acc=GSE21885

^3^www.geneontology.org

^4^http://www.transplant-tolerance.org.uk/

## References

[1] Aversa F Tabilio A Velardi A Cunningham I Terenzi A Falzetti F Treatment of high-risk acute leukemia with T-cell-depleted stem cells from related donors with one fully mismatched HLA haplotype. N Engl J Med (1998) 339(17):1186–93 10.1056/NEJM199810223391702 9780338

[2] Aversa F Terenzi A Tabilio A Falzetti F Carotti A Ballanti S Full haplotype-mismatched hematopoietic stem-cell transplantation: a phase II study in patients with acute leukemia at high risk of relapse. J Clin Oncol (2005) 23(15):3447–54 10.1200/JCO.2005.09.117 15753458

[3] Ciceri F Labopin M Aversa F Rowe JM Bunjes D Lewalle P A survey of fully haploidentical hematopoietic stem cell transplantation in adults with high-risk acute leukemia: a risk factor analysis of outcomes for patients in remission at transplantation. Blood (2008) 112(9):3574–81 10.1182/blood-2008-02-140095 18606875

[4] Lang P Handgretinger R Haploidentical SCT in children: an update and future perspectives. Bone Marrow Transplant (2008) 42(Suppl 2):S54–9 10.1038/bmt.2008.285 18978746

[5] Cavazzana-Calvo M Andre-Schmutz I Dal Cortivo L Neven B Hacein-Bey-Abina S Fischer A Immune reconstitution after haematopoietic stem cell transplantation: obstacles and anticipated progress. Curr Opin Immunol (2009) 21(5):544–8 10.1016/j.coi.2009.08.001 19766472

[6] Seggewiss R Einsele H Immune reconstitution after allogeneic transplantation and expanding options for immunomodulation: an update. Blood (2010) 115(19):3861–8 10.1182/blood-2009-12-234096 20215642

[7] Lewalle P Triffet A Delforge A Crombez P Selleslag D De Muynck H Donor lymphocyte infusions in adult haploidentical transplant: a dose finding study. Bone Marrow Transplant (2003) 31(1):39–44 10.1038/sj.bmt.1703779 12621505

[8] Culme-Seymour EJ Davie NL Brindley DA Edwards-Parton S Mason C A decade of cell therapy clinical trials (2000-2010). Regen Med (2012) 7(4):455–62 10.2217/rme.12.45 22817619

[9] Riddell SR Jensen MC June CH Chimeric antigen receptor – modified T cells: clinical translation in stem cell transplantation and beyond. Biol Blood Marrow Transplant (2013) 19(1 Suppl):S2–5 10.1016/j.bbmt.2012.10.021 23085599 PMC3654529

[10] Ciceri F Bonini C Stanghellini MT Bondanza A Traversari C Salomoni M Infusion of suicide-gene-engineered donor lymphocytes after family haploidentical haemopoietic stem-cell transplantation for leukaemia (the TK007 trial): a non-randomised phase I-II study. Lancet Oncol (2009) 10(5):489–500 10.1016/S1470-2045(09)70074-9 19345145

[11] Di Stasi A Tey SK Dotti G Fujita Y Kennedy-Nasser A Martinez C Inducible apoptosis as a safety switch for adoptive cell therapy. N Engl J Med (2011) 365(18):1673–83 10.1056/NEJMoa1106152 22047558 PMC3236370

[12] Porter DL Levine BL Kalos M Bagg A June CH Chimeric antigen receptor-modified T cells in chronic lymphoid leukemia. N Engl J Med (2011) 365(8):725–33 10.1056/NEJMoa1103849 21830940 PMC3387277

[13] Di Ianni M Falzetti F Carotti A Terenzi A Castellino F Bonifacio E Tregs prevent GVHD and promote immune reconstitution in HLA-haploidentical transplantation. Blood (2011) 117(14):3921–8 10.1182/blood-2010-10-311894 21292771

[14] Brunstein CG Miller JS Cao Q McKenna DH Hippen KL Curtsinger J Infusion of ex vivo expanded T regulatory cells in adults transplanted with umbilical cord blood: safety profile and detection kinetics. Blood (2011) 117(3):1061–70 10.1182/blood-2010-07-293795 20952687 PMC3035067

[15] Edinger M Hoffmann P Regulatory T cells in stem cell transplantation: strategies and first clinical experiences. Curr Opin Immunol (2011) 23(5):679–84 10.1016/j.coi.2011.06.006 21802270

[16] Bacchetta R Bigler M Touraine JL Parkman R Tovo PA Abrams J High levels of interleukin 10 production in vivo are associated with tolerance in SCID patients transplanted with HLA mismatched hematopoietic stem cells. J Exp Med (1994) 179(2):493–502 10.1084/jem.179.2.493 7905018 PMC2191349

[17] Groux H O’Garra A Bigler M Rouleau M Antonenko S de Vries JE A CD4+ T-cell subset inhibits antigen-specific T-cell responses and prevents colitis. Nature (1997) 389(6652):737–42 10.1038/39614 9338786

[18] Battaglia M Stabilini A Draghici E Gregori S Mocchetti C Bonifacio E Rapamycin and interleukin-10 treatment induces T regulatory type 1 cells that mediate antigen-specific transplantation tolerance. Diabetes (2006) 55(1):40–9 10.2337/diabetes.55.01.06.db05-0613 16380475

[19] Serafini G Andreani M Testi M Battarra M Bontadini A Biral E Type 1 regulatory T cells are associated with persistent split erythroid/lymphoid chimerism after allogeneic hematopoietic stem cell transplantation for thalassemia. Haematologica (2009) 94(10):1415–26 10.3324/haematol.2008.003129 19608686 PMC2754958

[20] Gagliani N Jofra T Stabilini A Valle A Atkinson M Roncarolo MG Antigen-specific dependence of Tr1-cell therapy in preclinical models of islet transplant. Diabetes (2010) 59(2):433–9 10.2337/db09-1168 19934002 PMC2809952

[21] Gagliani N Jofra T Valle A Stabilini A Morsiani C Gregori S Transplant tolerance to pancreatic islets is initiated in the graft and sustained in the spleen. Am J Transplant (2013) 13(8):1963–75 10.1111/ajt.12333 23834659 PMC3869180

[22] Gagliani N Magnani CF Huber S Gianolini ME Pala M Licona-Limon P Coexpression of CD49b and LAG-3 identifies human and mouse T regulatory type 1 cells. Nat Med (2013) 19(6):739–46 10.1038/nm.3179 23624599

[23] Magnani CF Alberigo G Bacchetta R Serafini G Andreani M Roncarolo MG Killing of myeloid APCs via HLA class I, CD2 and CD226 defines a novel mechanism of suppression by human Tr1 cells. Eur J Immunol (2011) 41(6):1652–62 10.1002/eji.201041120 21469116 PMC3116154

[24] Groux H Bigler M de Vries JE Roncarolo MG Interleukin-10 induces a long-term antigen-specific anergic state in human CD4+ T cells. J Exp Med (1996) 184(1):19–29 10.1084/jem.184.1.19 8691133 PMC2192687

[25] Bacchetta R Gregori S Serafini G Sartirana C Schulz U Zino E Molecular and functional characterization of allogantigen-specific anergic T cells suitable for cell therapy. Haematologica (2010) 95(12):2134–43 10.3324/haematol.2010.025825 20713457 PMC2995573

[26] Gregori S Tomasoni D Pacciani V Scirpoli M Battaglia M Magnani CF Differentiation of type 1 T regulatory (Tr1) cells by tolerogenic DC-10 requires the IL-10-dependent ILT4/HLA-G pathway. Blood (2010) 116(6):935–44 10.1182/blood-2009-07-234872 20448110

[27] Schwartz RH T cell anergy. Annu Rev Immunol (2003) 21:305–34 10.1146/annurev.immunol.21.120601.141110 12471050

[28] Zeller JC Panoskaltsis-Mortari A Murphy WJ Ruscetti FW Narula S Roncarolo MG Induction of CD4+ T cell alloantigen-specific hyporesponsiveness by IL-10 and TGF-beta. J Immunol (1999) 163(7):3684–91 10490963

[29] Gregori S Roncarolo MG Bacchetta R Methods for in vitro generation of human type 1 regulatory T cells. Methods Mol Biol (2011) 677:31–46 10.1007/978-1-60761-869-0_3 20941601

[30] Guihot A Dupin N Marcelin AG Gorin I Bedin AS Bossi P Low T cell responses to human herpesvirus 8 in patients with AIDS-related and classic Kaposi sarcoma. J Infect Dis (2006) 194(8):1078–88 10.1086/507648 16991082

[31] Zhang W Caspell R Karulin AY Ahmad M Haicheur N Abdelsalam A ELISPOT assays provide reproducible results among different laboratories for T-cell immune monitoring – even in hands of ELISPOT-inexperienced investigators. J Immunotoxicol (2009) 6(4):227–34 10.3109/15476910903317546 19908941

[32] Brouard S Dupont A Giral M Louis S Lair D Braudeau C Operationally tolerant and minimally immunosuppressed kidney recipients display strongly altered blood T-cell clonal regulation. Am J Transplant (2005) 5(2):330–40 10.1111/j.1600-6143.2004.00700.x 15643993

[33] Pannetier C Cochet M Darche S Casrouge A Zoller M Kourilsky P The sizes of the CDR3 hypervariable regions of the murine T-cell receptor beta chains vary as a function of the recombined germ-line segments. Proc Natl Acad Sci U S A (1993) 90(9):4319–23 10.1073/pnas.90.9.4319 8483950 PMC46498

[34] Sebille F Gagne K Guillet M Degauque N Pallier A Brouard S Direct recognition of foreign MHC determinants by naive T cells mobilizes specific Vbeta families without skewing of the complementarity-determining region 3 length distribution. J Immunol (2001) 167(6):3082–8 11544292 10.4049/jimmunol.167.6.3082

[35] Edgar R Domrachev M Lash AE Gene Expression Omnibus: NCBI gene expression and hybridization array data repository. Nucleic Acids Res (2002) 30(1):207–10 10.1093/nar/30.1.207 11752295 PMC99122

[36] Ernst J Bar-Joseph Z STEM: a tool for the analysis of short time series gene expression data. BMC Bioinformatics (2006) 7:191 10.1186/1471-2105-7-191 16597342 PMC1456994

[37] Saeed AI Sharov V White J Li J Liang W Bhagabati N TM4: a free, open-source system for microarray data management and analysis. Biotechniques (2003) 34(2):374–8 12613259 10.2144/03342mt01

[38] Sagoo P Perucha E Sawitzki B Tomiuk S Stephens DA Miqueu P Development of a cross-platform biomarker signature to detect renal transplant tolerance in humans. J Clin Invest (2010) 120(6):1848–61 10.1172/JCI39922 20501943 PMC2877932

[39] Wieczorek G Asemissen A Model F Turbachova I Floess S Liebenberg V Quantitative DNA methylation analysis of FOXP3 as a new method for counting regulatory T cells in peripheral blood and solid tissue. Cancer Res (2009) 69(2):599–608 10.1158/0008-5472.CAN-08-2361 19147574

[40] Newell KA Asare A Kirk AD Gisler TD Bourcier K Suthanthiran M Identification of a B cell signature associated with renal transplant tolerance in humans. J Clin Invest (2010) 120(6):1836–47 10.1172/JCI39933 20501946 PMC2877933

[41] Aversa F Martelli MF Transplantation of haploidentically mismatched stem cells for the treatment of malignant diseases. Spring Semin Immunopathol (2004) 26(1–2):155–68 10.1007/s00281-004-0161-7 15378270

[42] Grossman WJ Verbsky JW Barchet W Colonna M Atkinson JP Ley TJ Human T regulatory cells can use the perforin pathway to cause autologous target cell death. Immunity (2004) 21(4):589–601 10.1016/j.immuni.2004.09.002 15485635

[43] Grossman WJ Verbsky JW Tollefsen BL Kemper C Atkinson JP Ley TJ Differential expression of granzymes A and B in human cytotoxic lymphocyte subsets and T regulatory cells. Blood (2004) 104(9):2840–8 10.1182/blood-2004-03-0859 15238416

[44] Roncarolo MG Battaglia M Regulatory T-cell immunotherapy for tolerance to self antigens and alloantigens in humans. Nat Rev Immunol (2007) 7(8):585–98 10.1038/nri2138 17653126

[45] Riley JL June CH Blazar BR Human T regulatory cell therapy: take a billion or so and call me in the morning. Immunity (2009) 30(5):656–65 10.1016/j.immuni.2009.04.006 19464988 PMC2742482

[46] Hutchinson JA Riquelme P Sawitzki B Tomiuk S Miqueu P Zuhayra M Cutting edge: immunological consequences and trafficking of human regulatory macrophages administered to renal transplant recipients. J Immunol (2011) 187(5):2072–8 10.4049/jimmunol.1100762 21804023

[47] Dodero A Carniti C Raganato A Vendramin A Farina L Spina F Haploidentical stem cell transplantation after a reduced-intensity conditioning regimen for the treatment of advanced hematologic malignancies: posttransplantation CD8-depleted donor lymphocyte infusions contribute to improve T-cell recovery. Blood (2009) 113(19):4771–9 10.1182/blood-2008-10-183723 19211934

[48] Amrolia PJ Muccioli-Casadei G Huls H Adams S Durett A Gee A Adoptive immunotherapy with allodepleted donor T-cells improves immune reconstitution after haploidentical stem cell transplantation. Blood (2006) 108(6):1797–808 10.1182/blood-2006-02-001909 16741253 PMC1895537

[49] Guinan EC Boussiotis VA Neuberg D Brennan LL Hirano N Nadler LM Transplantation of anergic histoincompatible bone marrow allografts. N Engl J Med (1999) 340(22):1704–14 10.1056/NEJM199906033402202 10352162

[50] Davies JK Gribben JG Brennan LL Yuk D Nadler LM Guinan EC Outcome of alloanergized haploidentical bone marrow transplantation after ex vivo costimulatory blockade: results of 2 phase 1 studies. Blood (2008) 112(6):2232–41 10.1182/blood-2008-03-143636 18617635 PMC2532801

[51] Desreumaux P Foussat A Allez M Beaugerie L Hebuterne X Bouhnik Y Safety and efficacy of antigen-specific regulatory T-cell therapy for patients with refractory Crohn’s disease. Gastroenterology (2012) 143(5):e1–2 10.1053/j.gastro.2012.07.116 22885333

